# Pluripotent nontumorigenic multilineage differentiating stress enduring cells (Muse cells): a seven-year retrospective

**DOI:** 10.1186/s13287-017-0674-3

**Published:** 2017-10-18

**Authors:** Samantha C. Fisch, María L. Gimeno, Julia D. Phan, Ariel A. Simerman, Daniel A. Dumesic, Marcelo J. Perone, Gregorio D. Chazenbalk

**Affiliations:** 1 Department of Obstetrics and Gynecology, David Geffen School of Medicine, The University of California, 10833 Le Conte Ave, Box 951740, Los Angeles, CA 90095-1740 USA; 2 Instituto de Investigación en Biomedicina de Buenos Aires (IBioBA)—CONICET—Partner Institute of the Max Planck Society, Polo Científico Tecnológico, Godoy Cruz 2390, C1425FQD Buenos Aires, Argentina

**Keywords:** Adult pluripotent stem cells, Muse cells, Cellular stress, Nontumorigenic, Quiescence, High homing capacity, Regenerative medicine

## Abstract

Multilineage differentiating stress enduring (Muse) cells, discovered in the spring of 2010 at Tohoku University in Sendai, Japan, were quickly recognized by scientists as a possible source of pluripotent cells naturally present within mesenchymal tissues. Muse cells normally exist in a quiescent state, singularly activated by severe cellular stress in vitro and in vivo. Muse cells have the capacity for self-renewal while maintaining pluripotent cell characteristics indicated by the expression of pluripotent stem cell markers. Muse cells differentiate into cells representative of all three germ cell layers both spontaneously and under media-specific induction. In contrast to embryonic stem and induced pluripotent stem cells, Muse cells exhibit low telomerase activity, a normal karyotype, and do not undergo tumorigenesis once implanted in SCID mice. Muse cells efficiently home into damaged tissues and differentiate into specific cells leading to tissue regeneration and functional recovery as described in different animal disease models (i.e., fulminant hepatitis, muscle degeneration, skin ulcers, liver cirrhosis, cerebral stroke, vitiligo, and focal segmental glomerulosclerosis). Circulating Muse cells have been detected in peripheral blood, with higher levels present in stroke patients during the acute phase. Furthermore, Muse cells have inherent immunomodulatory properties, which could contribute to tissue generation and functional repair in vivo. Genetic studies in Muse cells indicate a highly conserved cellular mechanism as seen in more primitive organisms (yeast, *Saccharomyces cerevisiae*, *Caenorhabditis elegans*, *chlamydomonas*, *Torpedo californica*, *drosophila*, etc.) in response to cellular stress and acute injury. This review details the molecular and cellular properties of Muse cells as well as their capacity for tissue repair and functional recovery, highlighting their potential for clinical application in regenerative medicine.

**Electronic supplementary material:**

The online version of this article (10.1186/s13287-017-0674-3) contains supplementary material, which is available to authorized users.

## Background

### Gold standard pluripotent stem cells: embryonic and induced pluripotent stem cells

Regenerative medicine is a cross-disciplinary field that incorporates stem cell-based therapy, tissue production and repair, and disease modeling [1]. Despite many efforts over the last 15 years, most stem cell trials remain clinically stagnant in their early phases [2]. The discovery of embryonic stem (ES) cells in the late twentieth century introduced a promising cell population with an unrestrained proliferative capacity to differentiate into mesenchymal, ectodermal, and endodermal lineages [3, 4]. Ethical issues quickly arrested their upward climb. Furthermore, ES cells have been shown to display teratoma formation and immunorejection following transplantation [5]. These insurmountable impediments render ES cells an unrealistic contributor to the field of regenerative medicine.

Induced pluripotent stem (iPS) cells proposed a solution to the ethical concerns that hindered ES cells. Reprogramming adult somatic cells for intended reimplantation with induced expression of transcription factors generated a population of pluripotent stem cells that resolved the issue of immunorejection. Questions surrounding iPS cell carcinogenicity and teratoma formation in vivo as a result of unbridled cell proliferation, however, remain unresolved, precluding them from clinical application [6]. The first and only trial performed in Japan in 2015 came to a screeching halt just after the first patient had received iPS cell treatment due to regulatory issues surrounding the detection of harmful mutations [7].

### Potential pluripotency of adult stem cells

In the last decade, the scientific community has continued the collective effort to uncover a population of pluripotent stem cells lacking tumorigenic properties for utilization in cell-based therapy. In 2002, a population of multipotent adult progenitor cells (MAPC) was reported to be both pluripotent and nontumorigenic [8]. However, these results have yet to be reproduced. Other populations of cells isolated from bone marrow, termed very small embryonic-like stem cells (VSELs) and human marrow-isolated adult multilineage inducible (MIAMI) cells, have been shown to exhibit nontumorigenic and pluripotent properties [8, 9]. However, these stem cell populations remain controversial, as cell lines such as VSELs have shown evidence of aneuploidy [10]. Furthermore, other mesenchymal stem cells (MSCs) isolated from umbilical cord blood, referred to as unrestricted somatic stem cells (USSCs), show epigenetic evidence of an intermediate pluripotent phenotype while lacking expression of established pluripotent markers [11]. Another population of pluripotent stem cells was said to be generated through stimulus-triggered acquisition of pluripotency (STAP) by means of acidic exposure with leukemia inhibitory factor (LIF) to splenic CD45^+^ lymphocytes. However, many independent investigators have failed to reproduce this methodology, and all publications on STAP cells have since been repudiated. The original paper was retracted by its authors.

### Mesenchymal stem cells and pluripotency

Mesenchymal tissue is vastly connected to the immune system and contributes to inflammatory mechanisms during tissue repair [12]. MSCs are a naturally occurring population of nontumorigenic stem cells widely accepted as a multipotent heterogeneous cell population. However, it has been found that MSCs undergo triploblastic differentiation at low frequencies [13]. Several theories have surfaced surrounding the reason why MSCs exhibit a low degree of differentiation into other germ lineages. MSCs constitute a heterogeneous population of cells including endothelial cells and fibroblasts [13]. Some researchers hypothesize that there could be several kinds of stem cells within the heterogeneous MSC population that have different germ lineage proliferative capacities. Others have speculated that a single cell population exists with the capacity to differentiate into all three germlines [14].

## Main text

### The discovery of Muse cells

Multilineage differentiating stress enduring (Muse) cells were first isolated from bone marrow (BM) aspirates under long-term trypsin incubation by Dr Dezawa’s team in 2010. Muse cells show expression of cells double positive for CD105 (a marker of MSCs) and SSEA3 (a marker of ES cells) [14]. Muse cells comprise a small population of MSCs (e.g., BM-MSCs (1–2%)) [14–16], in dermal fibroblastic tissue (5%) [17], and in adipose tissue [18–22]. Therefore, it is possible to speculate that the unusually low frequency of pluripotent cell differentiation observed in MSCs is due to the presence of Muse cells within the total MSC population [23].

Muse cells form characteristic cell clusters in vitro, termed “M clusters” (Additional file 1 [22]), resembling the formation of embryonic and iPS cells [24]. Muse cells express pluripotent stem cell markers and self-renewal through several generation passages [14, 16, 18, 21, 22, 25, 26].



**Additional file 1:** is a video showing Muse-AT cells spontaneously form cell clusters within 24 hours (Reproduced from supplemental online Video 1 in Gimeno et al*.* [22] under CC-BY license). (MP4 53529 kb)


A defining characteristic of Muse cells is their potential for triploblastic differentiation from a single cell [15]. M clusters express pluripotent markers such as NANOG, Oct3/4, Par-4, Sox2, and TRA1-60 in addition to markers from mesodermal (NK2-5), endodermal (GATAφ, α-fetoprotein), and ectodermal (MAP2) cell lines spontaneously and under media-specific induction [20]. Seven independent groups have confirmed this inherent pluripotent phenotype over the last 7 years [14, 17, 18, 21, 22, 27, 28]. These groups have found Muse cells throughout different mesenchymal tissues including bone marrow, skin, and adipose tissue retaining the same pluripotent potential and Muse cell phenotype [14, 17, 18].

**Fig. 1:**
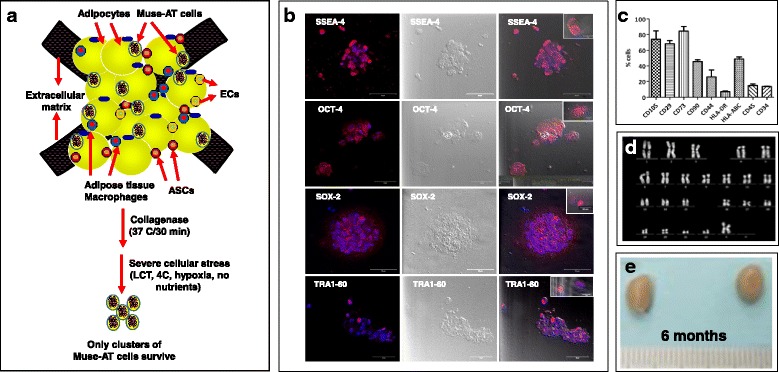
**a** Schematic of Muse-AT cell generation from lipoaspirate material. Different cellular components are present in adipose tissue (i.e., adipocytes, endothelial cells (ECs), adipose stem cells (ASCs), adipose tissue macrophages, and Muse-AT cells). Adipose tissue (lipoaspirate material) first exposed to collagenase for 30 minutes at 37 °C, and then for 12 hours under severe cellular stress conditions (i.e., long-term collagenase incubation, lack of nutrients, low temperature, and hypoxia). Only a cluster of Muse-AT cells survived such stress. **b** Expression of pluripotent stem cell markers SSEA4, Oct-4, Sox-2, and TRA1-6 in Muse-AT cells. **c** Expression of CD markers in Muse cells indicating an immunophenotype. **d** Evidence of a normal karyotype in Muse cells. **e** Muse-AT cells do not form teratomas after 6-month implantation in testis (right) in comparison with control, sham-injected testis (left). Muse-AT adipose tissue-derived multilineage differentiating stress enduring (**c** Reproduced from Figure 2 in Gimeno et al. [22] under CC-BY license) (**d** Reproduced from Figure 4 in Gimeno et al. [22] under CC-BY license) (**e** Reproduced from Figure 4 in Gimeno et al. [22] under CC-BY license)

Adipose tissue-derived Muse (Muse-AT) cells were first identified within lipoaspirate material exposed to severe cellular stress conditions such as long-term collagenase incubation, lack of nutrients, low temperature, and hypoxia [18] (Fig. 1a). Remarkably, cell expansion is unnecessary due to the large number of highly purified Muse-AT cells (250,000–500,000 cells/g of lipoaspirate material obtained by this technique) [18, 22]. Muse-AT cells as well as those derived from goat skin fibroblasts can be expanded at least 10 times without altering their phenotype [28, 29].

Muse cells initially discovered by cell sorting from bone marrow aspirates leads to low yield of about 1% of Muse cells within the total population (8000 cells/ml initial culture) [14]. However, Muse-AT cells isolated by severe cellular stress greatly increase this yield capacity, providing potential for feasible a clinical dose of Muse cells in humans [18, 22].

Muse-AT cells are positive for SSEA3, Oct3/4, NANOG, and Sox2 expression [18]. Recently, another study has confirmed the existence of Muse-AT cells with expression of the pluripotent markers Oct4, TRA1-60, SSEA4, NANOG, and Sox2 and their ability to self-proliferate through five passages in vitro (Fig. 1b) [22]. Muse-AT cells spontaneously differentiate into mesodermal, endodermal, and ectodermal cell lineages with an efficiency of 23% (BODIPY^+^ adipocytes, SMA^+^ and MyoD^+^ myocytes), 20% (α-fetoprotein hepatocytes, pan keratin biliary cells), and 22% (MAP2^+^ neural-like cells) respectively [18, 22]. Muse-AT cells can differentiate into all three germline cells under specific induction differentiated medium with an efficiency of 82% mesenchymal, 75% endodermal, and 78% ectodermal [18].

Muse-AT cells express low levels of genes involved in cell proliferation as well as oncogenicity, which might account for their low proliferative performance and lack of tumorigenic activity [18]. Furthermore, Muse-AT cells retain expression of several CD (clusters of differentiation) markers (Fig. 1c) [18, 22]. Genetic studies across several species (yeast, *Saccharomyces cerevisiae*, *Caenorhabditis elegans*, *chlamydomonas*, *Torpedo californica*, *drosophila*, etc.) display expression of genes related to cell death and survival, indicating a highly conserved cellular mechanism linked to cellular stress and acute injury. This suggests that Muse cells could play an evolutionary role in relation to cell survival under conditions of severe cellular stress [18, 20, 30].

### Nontumorigenic activity of Muse cells

The dual nature of pluripotency and tumorigenicity remains the prominent issue in identifying a cell population best suited to cell-based regenerative therapy. California’s Stem Cell agency along with other professional organizations define pluripotency as a cell’s potential to differentiate or take on the fates of all 200 different cell types [31]. However, several groups still use in-vivo teratoma formation as a reliable method of pluripotency determination [32]. The stringent mandatory criteria evaluates pluripotency by in vivo formation of teratomas would exclude stem cell populations exhibiting triploblastic differentiation capability. The existance of pluripotent stem cells without teratogenic activity challenges the current paradigm that ES cells and iPS are the only pluripotent stem cells available to be used in regenerative medicine.

Muse cells retain a stable karyotype in culture (Fig. 1d) and low telomerase activity [14, 15, 19, 20, 22]. Furthermore, Muse cells injected into the testes of mice do not show teratoma formation throughout a 6-month period **(**Fig. 1e), making them a much more attractive candidate than iPS cells for clinical application [14, 19, 20, 22, 23]. Cell expansion is unnecessary in the case of Muse-AT cells due to the large number obtained from lipoaspirates (250,000–500,000 cells/g of lipoaspirate material) [18, 22].

Muse cells show low expression of Lin28, a RNA binding protein gene involved in tumorigenesis and maintenance of pluripotency [14], in contrast to ES and iPS cells [1]. Muse cells also express high levels of Let-7, a microRNA responsible for regulating embryonic development, tumor suppression, and phenotypic differentiation, as a potential counteracting protective mechanism against Lin 28 and against tumorigenesis [18]. A steady decline in Lin28 is seen throughout embryonic development in combination with an increase in Let-7 expression, resulting in suppression of undifferentiated cell renewal while also stimulating cell differentiation [33]. A high Lin28/Let-7 ratio is exhibited in iPS and ES cells, likely responsible for their tumorigenic capability in vivo [33], while Muse cells show a high Let7/Lin28 ratio [15, 19]. We do not yet know whether altering the Let7/Lin28 ratio would render Muse cells to become tumorigenic, but we can predict that decreasing the Let7/Lin28 ratio may push Muse cells to grow at an accelerated pace and exhibit similar tumorigenic activity as seen in iPS and ES cells [19, 33].

In contrast to ES and iPS cells, Muse cells exhibit few epigenetic modifications. Muse cells display an increased methylation in genes encoding for NANOG and Oct3/4 in comparison to iPS cells, which might explain their lower expression of pluripotent stem cell markers and suppression in teratogenic activity [15]. Muse cells therefore retain their plasticity while remaining nonteratogenic.

### Muse cells display intrinsic immunomodulatory properties

Muse cells were primarily identified in mesenchymal tissues. Connective tissue containing blood vessels carries immune cells that provide protective support against potential pathogens. Therefore, mesenchymal tissue-resident Muse cells may locally influence the immune response. There are strong data supporting the modulatory effects of MSCs in the innate and adaptive arms of the immune system. In this respect, it is understood that MSCs suppress T-cell proliferation and cytokine secretion and regulate Th1/Th2 balance in dominant Th1-driven diseases and animal model of diseases [34, 35], induce regulatory T-cell (Treg) function [36], inhibit the maturation, activation, and antigen presentation of dendritic cells [37], and inhibit natural killer (NK) cell proliferation [38]. Similarly, pluripotent stem cells such as ES or iPS cells demonstrated high immunomodulatory potential by inhibition of effector T-lymphocyte proliferation including CD4^+^ or CD8^+^ T cells and NK cells [39]. The mechanisms by which MSCs promote regulatory functions in immune cells are not well understood. However, stem cell release of soluble immune modulatory mediators as well as contact between stem cells and immune cells could be an effective means to understand the mechanisms behind MSC immune modulatory capacity [40]. Several immunomodulators released by stem cells include hepatocyte growth factor (HGF), transforming growth factor-β1 (TGF-β1), indoleamine-pyrrole 2,3-dioxygenase (IDO), prostaglandin E2 (PGE2), heme oxygenase-1 (HO-1), interleukin-10 (IL-10), and nitric oxide (NO) [41]. Some of these modulatory agents can be induced and secreted after interaction between effector immune cells and stem cells. As an example, TGF-β seems to mediate beneficial effects and suppress a Th2-driven environment through i.v. administration of MSCs in a mouse model of ragweed-induced asthma [42].

Regarding Muse cells, the first evidence showing their immunomodulatory capacity was recently reported by Gimeno et al*.* and Alessio et al. [22, 43]. Muse-AT cells showed a potent immunomodulatory activity as indicated by a decrease in proinflammatory TNF-α in LPS-stimulated RAW 264.7 cells (a mouse macrophage-like cell line) and freshly isolated peritoneal murine macrophages incubated with Muse-AT conditioned media in vitro. Furthermore, Muse-AT cell conditioned media diminished the antigen-specific stimulation of Th1-type cytokines (IFN-γ and TNF-α). In both T cells and macrophages, Muse-AT cell conditioned media favored the secretion of IL-10, a cytokine associated with regulatory activity when expressed in immune cells [22].

**Fig. 2:**
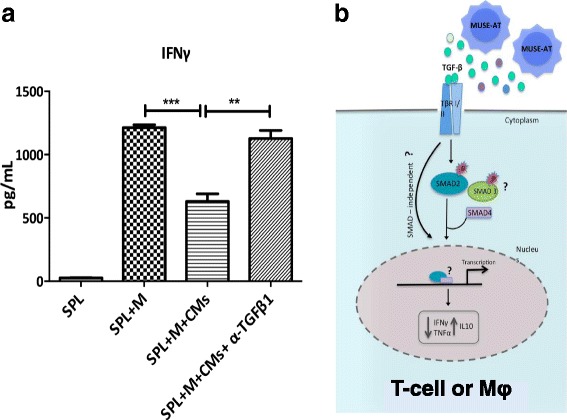
**a** TGF-β1 signaling blockade on IFN-γ secretion. Using a neutralizing monoclonal anti-TGF-β1, the inhibitory action on IFN-γ secretion was abolished in antigen (M)-specific stimulation of T cells. T cells were obtained from transgenic NOD BDC2.5 mice. Results representative of five separate experiments (Gimeno et al., unpublished data, 2017). **b** Putative intracellular signaling of TGF-β1 secreted by Muse-AT cells on T lymphocytes and macrophages. IFN-γ interferon gamma, IL interleukin, Muse-AT adipose tissue-derived multilineage differentiating stress enduring, TGF-β1 transforming growth factor-β1

TGF-β has been indicated as a key immunosuppressive cytokine under specific circumstances and is highly expressed in Muse-AT cells [22]. Indeed, using a small molecule inhibitor of the type I TGF-β receptor, the immune regulatory activity of Muse-AT conditioned media on T cells and macrophages responses was reversed almost completely [22]. To analyze further the production of TGF-β by Muse-AT cells, the use of a neutralizing anti-TGF-β1 in the culture media of antigen-specific stimulation of T lymphocytes reestablished IFN-γ secretion (unpublished data, Fig. 2a).

Moreover, Muse-AT cell conditioned media activate TGF-β1 signaling pathway as evidenced by high intracellular levels of pSMAD2 in antigen-specific T-lymphocyte stimulation [22]. However, whether there is a coexistence of SMAD-independent signaling to control T-cell and macrophage functions by Muse-AT cells remains to be elucidated (Fig. 2b).

### In-vivo effects of Muse cells in different animal models of human disease

Although overwhelming evidence has shown that Muse cells are a unique population of pluripotent nontumorigenic stem cells with regenerative potential, no clinical studies have since been conducted. All studies on Muse cells have so far been conducted through isolation of Muse cells derived from humans in several mesenchymal tissues that regenerate damaged tissues in different murine animal models. Since Muse cell discovery in 2010, many preclinical studies have been performed that could have the potential to impact the field of regenerative medicine and disease mitigation.

Liver disease affects one in every 10 Americans each year through cirrhosis, hepatitis, liver cancer, and other forms [44]. Muse cells could aid in the reversal of widespread liver damage and help to improve chronic liver disease. BM-derived Muse cells successfully differentiate into hepatocytes [16, 26]. Muse cells intravenously injected in a damaged liver mice model, intravenous Muse cells implant at the transection border of the hepatic tissue, and remain integrated, while non-Muse counterparts (defined by SSEA3^–^ labeling) are not detected 4 weeks later [16]. Integration of Muse cells into the damaged liver tissue was heterogeneous. A robust amount of Muse cell integration was determined by human-Golgi^+^ cells (1.89 ± 0.65% of total cells/mm^2^) detected in the samples injected with Muse cells [16]. This cellular integration was 48-fold higher than in samples injected with non-Muse cells (0.04 ± 0.08% total cells/mm^2^) [16]. Fluorescent labeling rules out the possibility of Muse cell fusion with previously established hepatic cells [16].

**Fig. 3:**
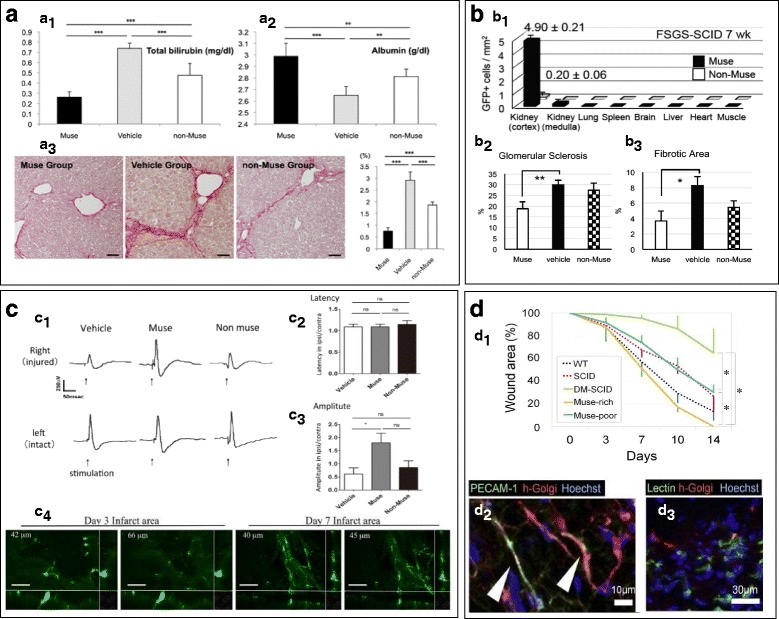
**A** Effect of Muse cells in damaged liver. Functional improvement shown in Muse cells by a decrease in bilirubin production, increase in albumin levels, and decrease in fibrotic tissues. **B** Effect of Muse cells in damaged kidney: (b_1_) detection of GFP(+) Muse cells distributed in different tissues after 7 weeks of injection in FSGS-SCID mice; (b_2_, b_3_) Muse cells show significant decrease in glomerular sclerosis as well as fibrotic areas. **C** Effect of Muse cells in damaged neural tissue: (c_1_) ipsilateral sensory cortex analysis in cerebral stroke-SCID mice after 84 days of Muse, non-Muse, and vehicle treatment; somatosensory evoked potentials show no effect in latency (c_2_) and significant increase in amplitude (c_3_) between Muse cells and vehicle controls; (c_4_) integration of GFP(+) Muse cells into neural tissue at days 3 and 7 display neurite-like cell formation. **D** Effect of Muse cells in diabetic skin ulcers: (d_1_) Muse-rich fraction shows significant reduction in percent wounded area in comparison with Muse-poor fraction at 14 days post implantation; (d_2_) Muse-rich fraction expresses PECAM-1 and isolectin (markers of dermis and vascular endothelial cells) in upper dermis at 14 days post implantation; (d_3_) Muse cells show negative expression of PECAM-1 and isolectin in middle and lower dermis 14 days post implantation. Muse multilineage differentiating stress enduring, ns not significant (**A** Reproduced with permission from Figure 4 in Iseki et al. [26]) **(**b_1_ Reproduced with permission from Figure 2 in Uchida et al. [46], License Number 4141730401653) (b_2_, b_3_ Reproduced with permission from Figure 6 in Uchida et al*.* [46], License Number 4141730401653) (c_1_–c_3_ Reproduced from Figure 6 in Uchida et al. [23] under CC-BY license) (c_4_ Reproduced from Figure 7 in Uchida et al. [23] under CC-BY license) (d_1_ Reproduced with permission from Figure 5 in Kinoshita et al. [21], License Number 4136900281603) (d_2_, d_3_ Reproduced with permission from Figure 7 in Kinoshita et al. [21], License Number 4136900281603)

Intravenous infusion of Muse cells in this damaged liver mice model shows homing and integration into localized inflammatory sites in the liver, while abstaining from integration in other tissues besides the lungs 2 weeks post intravenous induction [26]. Functional improvement has been measured by a significant decrease in bilirubin production, an increase in albumin levels, and a decrease in fibrotic tissues as compared to non-Muse controls [26] (Fig. 3A). Furthermore, 97% of Muse cells that incorporate in the liver differentiate into HepPar-1-positive cells expressing human-specific albumin without any evidence of cell fusion, showing that preimplantation induction is not necessary due to spontaneous differentiation of Muse cells in vivo [26].

Chronic kidney disease (CKD) has been on the rise since the 1980s, now reaching an overall prevalence of 14% in Americans with fewer than 20,000 transplants per year [45]. Focal segmental glomerulosclerosis (FSGS) is a common precursor to CKD characterized by extensive scarring and progressive glomerulosclerosis [46]. Recently a group of scientists constructed a rodent model resembling FSGS in immune-deficient SCID mice and BALB/c mice lacking concurrent immunosuppression [46]. In both FSGS mice models, intravenously injected Muse cells preferentially integrate into damaged glomeruli as shown by GFP(+) detection amounting to approximately 3.67 ± 0.26 Muse cells/mm^2^ and 4.90 ± 0.21 Muse cells/mm^2^ at 2 weeks and 7 weeks respectively in the kidney cortex, in contrast to non-Muse cells which mainly remain in the spleen and lung [46] (Fig. 3B b_1_). Cell fusion between Muse and host cells has been ruled out by fluorescent in-situ hybridization, and Muse cells are detected in the SCID mice for up to 7 weeks and in BALB/c mice for up to 5 weeks post implantation [46]. Despite the lack of immunosuppressants in the BALB/c model, functional improvement has been demonstrated through improved urine protein, plasma creatinine levels, and creatinine clearance at 5 weeks with no difference at 7 weeks [46]. In comparison to non-Muse and vehicle controls, Muse cells show significant reduction in glomerular sclerosis and interstitial fibrosis (Fig. 3B b_2_, b_3_) [46].

Strokes are the leading cause of serious long-term disability and cost the United States approximately $33 billion each year [47]. Preclinical studies have shown that Muse cells contribute to neural cell regeneration and functional recovery [23, 48, 49]. Human Muse cells are successfully mobilized from bone marrow into bloodstream circulation immediately following post-ischemic stroke [27]. While Muse cells are usually mobilized into circulation in low endogenous numbers, administering a pure population of Muse cells could potentially produce significant functional improvement following stroke through efficient cell regeneration [27]. Muse cells integrate into damaged neural tissue and express neuronal factors Tuj-1 and NeuN, with relative expression of 45.3% and 20.5% respectively, suggesting a spontaneous differentiation capacity in vivo [48]. Only Muse cells incorporate into the tissue post transplantation, exemplifying their graft survival under stressful conditions [48].

Moreover, Muse cells successfully integrate into areas of cerebrovascular damage due to their high resistance to cellular stress in hematoma presence [49]. When implanted in vivo, post cerebral hemorrhage, Muse cells exhibited an accelerated and significant recovery of motor skills in mice in comparison to non-Muse controls using the Morris water maze and motor function test [49]. Muse cells integrate into neural tissue and begin to extend neurite like outgrowths (Fig. 3C c_4_). Muse cells also integrate into the motor and sensory cortex and show functional improvement in the neural circuit as measured by a modified neurological severity score and rotarod test [23]. Hind-limb somatosensory evoked potentials exhibit significantly higher amplitude effects, indicating electrophysiological improvement, but only a nonsignificant trend towards a higher level of latency was found in Muse vs vehicle controls (Fig. 3C c_1_–c_3_) [23].

Muse cells derived from dermal scalp fibroblasts display a melanocyte phenotype through expression of melanocyte-specific antigen HMB45 following media-specific induction [17]. An inverse correlation was found between age and number of Muse cells isolated from different patients, but no difference in pluripotency expression was exhibited regardless of age [50]. This correlation between total Muse cell count and age provides a contributing factor to the age effect in relative healing time, especially in surface wounds. Muse-AT cells also successfully differentiate into dermal cells and promote wound healing in diabetic skin ulcers (Fig. 3D d_1_) [21]. Immunohistochemistry studies confirm that human Golgi complex-positive Muse cells survive 14 days post implantation and differentiate into dermis and vascular endothelial cells through PECAM-1 and isolectin detection in the upper dermis (Fig. 3D d_2_) and into other cell types as indicated by negative PECAM-1 and isolectin in the lower and middle dermis (Fig. 3D d_3_) [21].

## Conclusions

Significant progress has occurred since Muse cells were introduced to the scientific community in 2010. Muse cells express classic pluripotency markers including SSEA-3, Oct 3/4, NANOG, Sox2, and Par-4, although at much lower levels than ES or iPS cells. Furthermore, Muse cells can differentiate to the three embryonic germ layers in vitro and in vivo without teratoma formation. Muse cells also exhibit a stable normal karyotype in culture, as indicated by their normal chromosome number and integrity. Because naïve Muse-AT cells exist in a quiescent state, they are intrinsically resilient to survival when transplanted back into the host organism. Muse cells successfully regenerate skin, muscle, liver, kidney, and neural damaged tissues showing high capacity of homing, integration into damaged tissues, replenishing new cells, and restoring tissue function as demonstrated in different animal disease models. Based on their unique qualities and vast potential, Muse cells are a very promising candidate for tissue regeneration and stem cell therapy.

## Abbreviations

BM-MSC: Bone marrow-derived mesenchymal stem cell
CKD: Chronic kidney disease
ES: Embryonic stem
FSGS: Focal segmental glomerulosclerosis
HGF: Hepatocyte growth factor
HO-1: Heme oxygenase-1
IDO: Indoleamine-pyrrole 2,3-dioxygenase
IFN-γ: Interferon gamma
IL-10: Interleukin-10
iPS: Induced pluripotent stem
LIF: Leukemia inhibitory factor
MAPC: Multipotent adult progenitor cells
MIAMI: Marrow-isolated adult multilineage inducible
MSC: Mesenchymal stem cell
Muse: Multilineage differentiating stress enduring
Muse-AT: Muse cells derived from adipose tissue
NK: Natural killer
NO: Nitric oxide
PGE2: Prostaglandin E2
STAP: Stimulus-triggered acquisition of pluripotency
TGF-β1: Transforming growth factor-β1
TNF-α: Tumor necrosis factor alpha
USSC: Unrestricted somatic stem cell
VSEL: Very small embryonic-like stem cell
